# Giant gastric lipossarcoma: case report and review of the literature

**DOI:** 10.1590/S1679-45082016RC3770

**Published:** 2016

**Authors:** Jacques Matone, Samuel Okazaki, Gabriel Naman Maccapani, Thiago Trolez Amancio, Renée Zon Filippi, Antonio Luiz de Vasconcellos Macedo

**Affiliations:** 1 Hospital Israelita Albert Einstein, São Paulo, SP, Brazil.

**Keywords:** Liposarcoma/diagnosis, Liposarcoma/surgery, Stomach neoplasms/diagnosis, Biopsy, Gastrectomy, Case reports

## Abstract

Liposarcoma is one of the most common soft tissue sarcomas in adults, occurring in 15 to 20% of all patients with sarcoma. Primary liposarcoma of the stomach is rare. We report a case of patient with giant gastric liposarcoma who underwent surgery after a gastrointestinal bleeding. Preoperative hystopathological diagnosis was not established, even after three biopsy attempts. We discuss differential diagnosis, genetic causes, diagnosis strategies and treatment.

## RESUMO

O lipossarcoma é um tipo comum de sarcomas em adultos, com incidência entre 15 e 20% entre os sarcomas. No entanto, o acometimento do estômago é raro. Relatamos um caso de um lipossarcoma primário gástrico gigante com apresentação clínica de hemorragia digestiva. Foi submetido a tratamento cirúrgico sem diagnóstico definitivo, apesar de três biópsias realizadas. Revisamos diagnósticos diferenciais, influência genética e estratégias diagnósticas e terapêuticas.

## INTRODUCTION

Liposarcoma is one of the most common soft tissue sarcomas in adults, occurring in 15 to 20% of all patients with sarcoma. The hallmark of liposarcoma pathophysiology is the immature fat cells or lipoblasts. This disease usually affects the limbs, retroperitoneum and trunk. Viscera are rarely involved. Primary liposarcoma of the stomach is exceptionally rare and less than 15 cases have been reported to date, since the first case report in 1941.^(1)^

Gastric liposarcomas are usually characterized by an exophytic growth tumor adherent to the gastric wall. Almost 75% of gastric liposarcomas are located in the antrum and they are usually of submucosal origin.

It is often misdiagnosed due to its rarity and absence of symptoms. Diagnosis is confirmed only after histopathological examination of surgical specimen. Standard preoperative biopsies are often inadequate because of the submucosal location of the tumor.^(2)^

We report a recent case of liposarcoma of the stomach, review current literature and discuss the differential diagnoses.

## CASE REPORT

A 76-year-old man presented to our emergency service with a gastrointestinal bleeding. His laboratory exams revealed no anemia and no coagulation disturbances.

The patient had a medical history of acute diverticulitis four years before, and on that time the computerized tomography (CT) scan showed a fatty tissue tumor of 5.0cm adjacent to the gastric wall. The endoscopic ultrasound performed with fine needle biopsy suggested only a lipoma. Because of comorbidities presented, such as morbid obesity, diabetes, dyslipidemia and systemic hypertension, surgery was not indicated because it appeared to be a benign lesion.

His familiar history was remarkable. His father had a gastric leyomiosarcoma operated on at the age of 61 year old, and he died of a giant retroperitoneal liposarcoma 20 years later. The patient sister also had a small bowel gastrointestinal stromal tumor (GIST), another soft tissue tumor, at the age of 68. Patient’s laboratory exams are described in table 1.

**Table 1:** Laboratory exams upon admission

Hemoglobin	15.4
Hematocryt	44.2
Leukocytes	9,870
Platelets	168.000
INR	1.0
CA 19.9	8.73
CEA	1.29
CA 15.3	5.1

**Figure 1:**
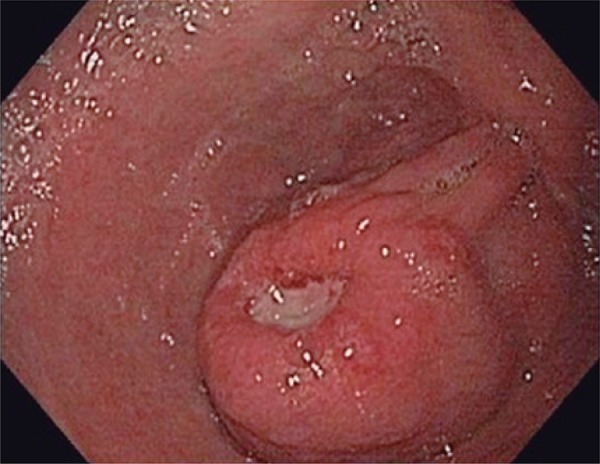
Ulcerated great curvature pre-pyloric lesion (8mm), with submucosal aspect, endoscopic view

The upper endoscopy revealed a soft (Figure 1), large, ulcerated, submucosal mass in the gastric antrum and the posterior gastric wall seemed to be compressed. Multiple biopsies were obtained but they were all superficial, therefore, showing unspecific inflammation of the gastric mucosa.

**Figure 2:**
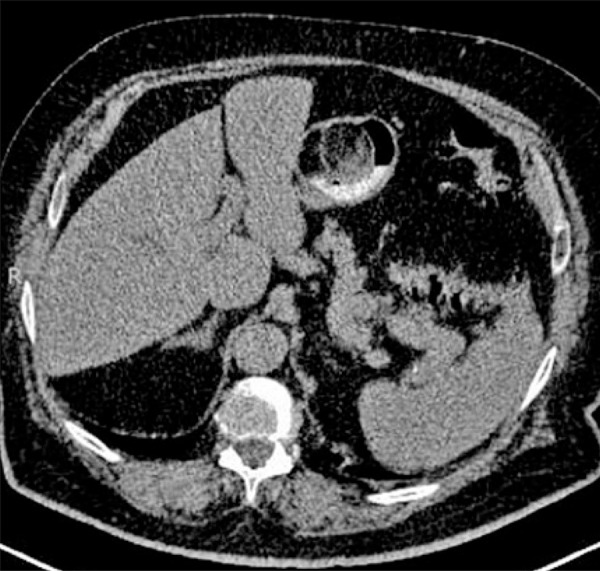
Computed tomography showing a well circumscribed mass with soft-tissue density and protruding into the gastric lumen

The abdominal (Figure 2) CT scan revealed a round, well circumscribed, low-attenuation, gastric antral mass, measuring approximately 7.5cm in diameter arising from the gastric lesser curvature and protruding beyond the gastric wall, which was in contact with the pancreas and gallbladder. No hepatic metastasis or nodal involvements were seen. Pre- and perioperative findings suggested a benign lipoma of the stomach wall. Pre-operative PET-C scan did not reveal any capitation.

The patient underwent a laparoscopic partial gastrectomy following gastroenteroanastomosis. Intra-operative pathology freezing study revealed free margins of the specimen. He had uneventful recovery and was discharged after 4 days.

The patient did not undergo any adjuvant treatment. A CT scan 6 months after the procedure showed no recurrence. The patient will remain in imaging follow-up for the next years.

### Hystopathological findings

The gross sections showed a large, well-circumscribed mass measuring 7.5 *versus* 7.0cm, arising from the gastric wall, ulcerating mucosa.

**Figure 3:**
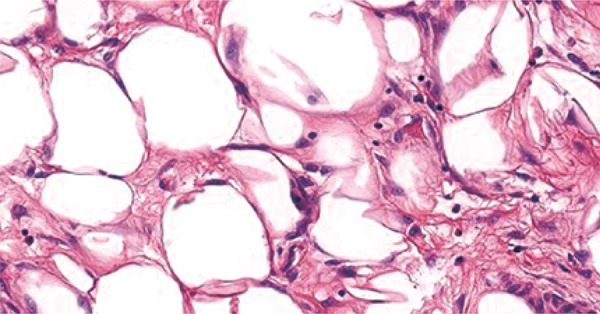
Lipoblats (hematoxylin and eosin)

**Figure 4:**
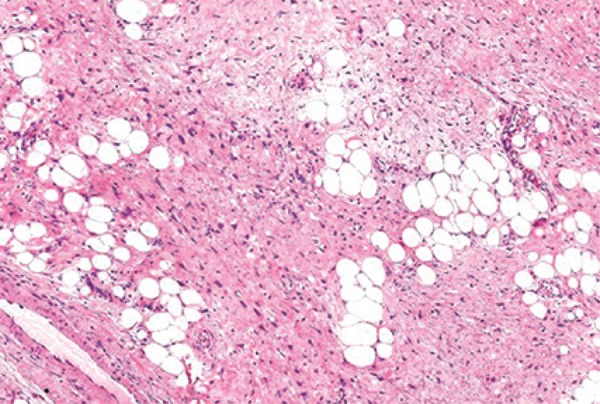
Adipose tissue and thick fibrous septa (hematoxylin and eosin)

**Figure 5:**
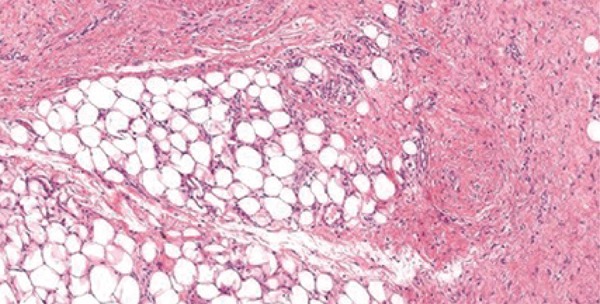
Adipose tissue and thick fibrous septa (hematoxylin and eosin)

The histopathological examination revealed a well-differentiated liposarcoma. The neoplasia was predominantly composed of mature adipocytes that varied in size and shape and had enlarged atypical hyperchromatic nuclei. Lipoblasts were sparsely distributed throughout the lesion. Thick hypercelular septa were seen (Figures 3 to 5). No mitoses were detected. There were no dedifferenciated or myxoid areas.

## DISCUSSION

Liposarcoma is a tumor more frequently found in adults with a peak incidence between the age 50 and 65 years. It is the most common soft tissue sarcoma appearing anywhere in the body.

Fatty tumors are rare in the gastrointestinal tract. Differentiating benign from malignant neoplasms is sometimes difficult because of morphologic features.

Liposarcoma is histologically defined as a tumor composed of lipoblasts. They are classified histologically, into five subtypes, each with its own unique characteristics and behaviors.

- Well-differentiated liposarcoma is the most common subtype and usually starts as a low grade tumor. Low grade tumor cells look much like normal fat cells under the microscope and tend to grow and change slowly.

- Myxoid liposarcoma is an intermediate to high grade tumor. Its cells look less normal under the microscope and may have a high grade component.

- Round cell mostly occurs on the limbs, with excessive proliferation of small rounded cells

- Pleomorphic liposarcoma is the rarest subtype and constitutes a high grade tumor with cells that look very different from normal cells.

- Dedifferentiated liposarcoma occurs when a low grade tumor changes, and the newer cells in the tumor are high grade.

Gastric liposarcomas originate due to proliferation of undifferentiated mesenchymal cells within the submucosa and the tunica muscularis layer of the stomach. Although 30% of well-differentiated liposarcomas present with local recurrence, metastasis is virtually never seen unless dedifferentiation occurs.

Overall mortality rates ranges from zero for atypical lipomatous tumor of the extremities to nearly 80 % for tumors occurring in the visceral sites and retroperitoneum.

Cytology is important in diagnosis since in adipose tissue tumors with fat less than 75% of the tumor volume, liposarcoma is the most likely diagnosis.^(3)^

When the tumor is large, there is a progressive tendency for the submucosal mass to extrude into the lumen, leading to traumatic and inflammatory changes and, therefore, resulting in necrosis, ulceration, and hemorrhage, as occurred with our patient.

The standard therapy is surgical excision. However, although data published is limited, surgical resection appears to be the best treatment modality with great increase of rates of disease-free and overall survival.

In difficult cases where complete resection is not possible or difficulty ensues in identifying the margin, *en* bloc debulking is the best option. Successful complete resection of retroperitoneal liposarcoma may increase the 5-year survival rate. To the best of our knowledge, there is currently no evidence that chemotherapy or radiotherapy improve survival rates.

Although the causes of soft tissue tumors are largely unknown, some environmental risk factors, such as ionizing radiation, immunosuppressive drugs, human immunodeficiency virus, and occupational exposure to vinyl chlorides, phenoxy-herbicides, arsenical pesticides, and dioxins, are known or suggested as risk factors.^(4)^

Our patient had a familial history of soft tissue tumors − a giant retroperitoneal liposarcoma on his father and an intestinal GIST on his sister, therefore, bringing the possibility of a genetic study. A small fraction of soft tissue tumors may be attributed to some rare hereditary cancer syndromes, including retinoblastoma,^(5)^ Li-Fraumeni syndrome^(6)^ neurofibromatosis type 1, and Gardner and Werner syndromes.^(7)^

The susceptibility genes for all these syndromes have been identified. Soft tissue tumors are characterized by frequent somatic chromosomal rearrangements.^(4,7)^

Differential diagnosis of gastric liposarcoma includes gastric stromal tumors, peritoneal carcinomatosis, peritoneal liposarcoma, carcinoma engulfing perivisceral fat, hepatic metastasis adjacent to the stomach, lymphoma and primary tumor of the omentum.^(8,9)^Recently, the endoscopic ultrasound is claimed to be the most useful diagnostic tool for these neoplasias that originate from the submucosa, and to exclude other diagnosis. However, further study for development of the method is necessary.^(10)^

## CONCLUSION

Because the benign or malignant nature of a submucosal lesion could be diagnosed with certainty, the gastric liposarcoma and the mesenchymal tumor of the stomach wall should be included in the differential diagnosis.
